# TGF-**β** signaling in myeloproliferative neoplasms contributes to myelofibrosis without disrupting the hematopoietic niche

**DOI:** 10.1172/JCI154092

**Published:** 2022-06-01

**Authors:** Juo-Chin Yao, Karolyn A. Oetjen, Tianjiao Wang, Haoliang Xu, Grazia Abou-Ezzi, Joseph R. Krambs, Salil Uttarwar, Eric J. Duncavage, Daniel C. Link

**Affiliations:** 1 Division of Oncology, Department of Medicine and; 2 Department of Pathology and Immunology, Washington University School of Medicine, St. Louis, Missouri, USA.

**Keywords:** Hematology, Oncology, Bone marrow, Fibrosis, Leukemias

## Abstract

Myeloproliferative neoplasms (MPNs) are associated with significant alterations in the bone marrow microenvironment that include decreased expression of key niche factors and myelofibrosis. Here, we explored the contribution of TGF-**β** to these alterations by abrogating TGF-**β** signaling in bone marrow mesenchymal stromal cells. Loss of TGF-**β** signaling in *Osx-Cre*–targeted MSCs prevented the development of myelofibrosis in both *MPL^W515L^* and *Jak2^V617F^* models of MPNs. In contrast, despite the absence of myelofibrosis, loss of TGF-**β** signaling in mesenchymal stromal cells did not rescue the defective hematopoietic niche induced by *MPL^W515L^*, as evidenced by decreased bone marrow cellularity*,* hematopoietic stem/progenitor cell number, and *Cxcl12* and *Kitlg* expression, and the presence of splenic extramedullary hematopoiesis. Induction of myelofibrosis by *MPL^W515L^* was intact in *Osx-Cre Smad4^fl/fl^* recipients, demonstrating that SMAD4-independent TGF-**β** signaling mediates the myelofibrosis phenotype. Indeed, treatment with a c-Jun N-terminal kinase (JNK) inhibitor prevented the development of myelofibrosis induced by *MPL^W515L^*. Together, these data show that JNK-dependent TGF-**β** signaling in mesenchymal stromal cells is responsible for the development of myelofibrosis but not hematopoietic niche disruption in MPNs, suggesting that the signals that regulate niche gene expression in bone marrow mesenchymal stromal cells are distinct from those that induce a fibrogenic program.

## Graphical abstract

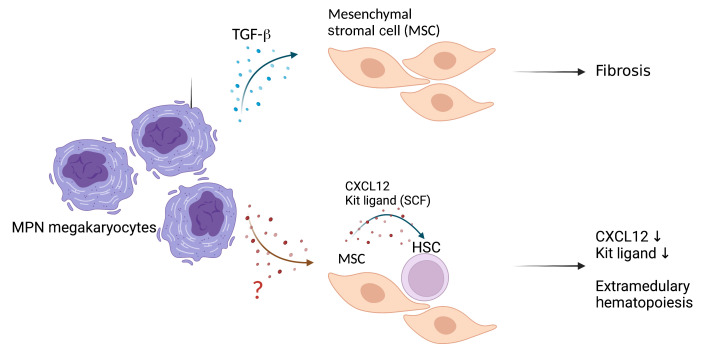

## Introduction

The bone marrow provides a specialized microenvironment that supports hematopoiesis. Hematopoietic niches in the bone marrow are dependent, in part, on the constitutive production of certain cytokines and chemokines by a heterogeneous population of stromal cells. For example, expression of Kit ligand (*Kitlg*) and CXCL12 by perivascular mesenchymal stromal cells (MSCs) plays an essential role in hematopoietic stem cell (HSC) maintenance (1–3). There are emerging data suggesting that malignant hematopoietic cells induce alterations in the bone marrow microenvironment that contribute to hematopoietic phenotypes (4). A better understanding of the interactions of malignant hematopoietic cells with bone marrow MSCs may identify novel targets for therapeutic intervention.

Myeloproliferative neoplasms (MPNs) are chronic myeloid malignancies characterized by overproduction of myeloid lineage cells (5). Activating mutations in genes impacting the JAK/STAT signaling pathway are the major causes of these cancers, including mutations in *JAK2*, *CALR*, and *MPL* (6). Alterations in the bone marrow microenvironment are a prominent feature of MPNs. Most striking is the development of myelofibrosis, which is characterized by extensive collagen deposition in the bone marrow and is associated with a poor prognosis (7, 8). Recent data show that RNA expression of key niche factors, including *Kitlg* and *Cxcl12*, is reduced in bone marrow MSCs from mouse models of MPNs and from patients with myelofibrosis (9–11). This is relevant, since studies by our group and others have shown that deleting these niche factors from stromal cells results in a loss of hematopoietic stem/progenitor cells (HSPCs) in the bone marrow and development of extramedullary hematopoiesis (1–3). There is also evidence that expression of CXCR4, the major receptor for CXCL12, is reduced on HSPCs in patients with MPNs, which would further reduce CXCL12 signaling (12). On the other hand, Migliaccio et al. reported that CXCL12 protein expression, as measured by immunostaining, is increased in the bone marrow of patients with MPNs and in the *Gata1^low^* mouse model of MPN (13). Thus, the impact of the MPN on bone marrow stromal niche factor expression (particularly CXCL12) remains unclear. Finally, there is evidence for altered bone metabolism in MPNs, with osteosclerosis reported in patients with myelofibrosis (14). Collectively, these alterations in the bone marrow microenvironment in MPNs are predicted to reduce its ability to support hematopoiesis. Indeed, extramedullary hematopoiesis and splenomegaly are common in MPNs.

There is evidence implicating inflammatory mediators in the development of myelofibrosis. In particular, increased production of TGF-β produced by megakaryocytes and monocytes is found in most patients with MPNs (15–19). Using a thrombopoietin (*TPO*) overexpression model of MPN, Chagraoui et al. showed that deletion of *Tgfb1* reduced but did not completely abrogate the development of myelofibrosis (20). Likewise, treatment with an inhibitor targeting TGFBR1 (ALK5) reduced myelofibrosis in mouse models of MPNs (21, 22). However, the role of TGF-β in the development of myelofibrosis remains controversial. In the aforementioned studies, although reduced, significant myelofibrosis remained despite inhibiting TGF-β signaling, raising the possibility that there are other mediators of myelofibrosis. Consistent with this possibility, Decker et al. showed that platelet-derived growth factor α (PDGFα) signaling in leptin receptor–positive (Lepr^+^) MSCs is required for the development of myelofibrosis (9). Moreover, Schneider et al. reported that CXCL4 signaling in Gli1^+^ MSCs contributes to myelofibrosis (10, 23). The cell of origin responsible for myelofibrosis is also controversial. As noted above, prior studies have implicated Lepr^+^ or Gli1^+^ MSCs as the target of inflammatory signaling leading to myelofibrosis (9, 10, 23). On the other hand, there are studies suggesting that monocyte-derived fibrocytes (24, 25) or endothelial cells (26) are the cell of origin.

In this study, we tested the hypothesis that TGF-β signaling in MSCs contributes to the development of myelofibrosis and hematopoietic niche disruption in MPNs. We show that genetic ablation of TGF-β signaling in MSCs, but not osteolineage cells, abrogates myelofibrosis. This effect is mediated by noncanonical c-Jun N-terminal kinase (JNK) signaling, and treatment of mice with a JNK inhibitor prevents the development of myelofibrosis. However, loss of TGF-β signaling in MSCs does not rescue the impaired hematopoietic niche function in MPNs, with persistent decreases in bone marrow cellularity, HSPC number, and *Cxcl12* and *Kitlg* expression, and development of extramedullary hematopoiesis.

## Results

### TGF-β signaling in MSCs is required for the efficient induction of myelofibrosis by MPL^W515L^ or Jak2^V617F^ in mice.

**Figure 1:**
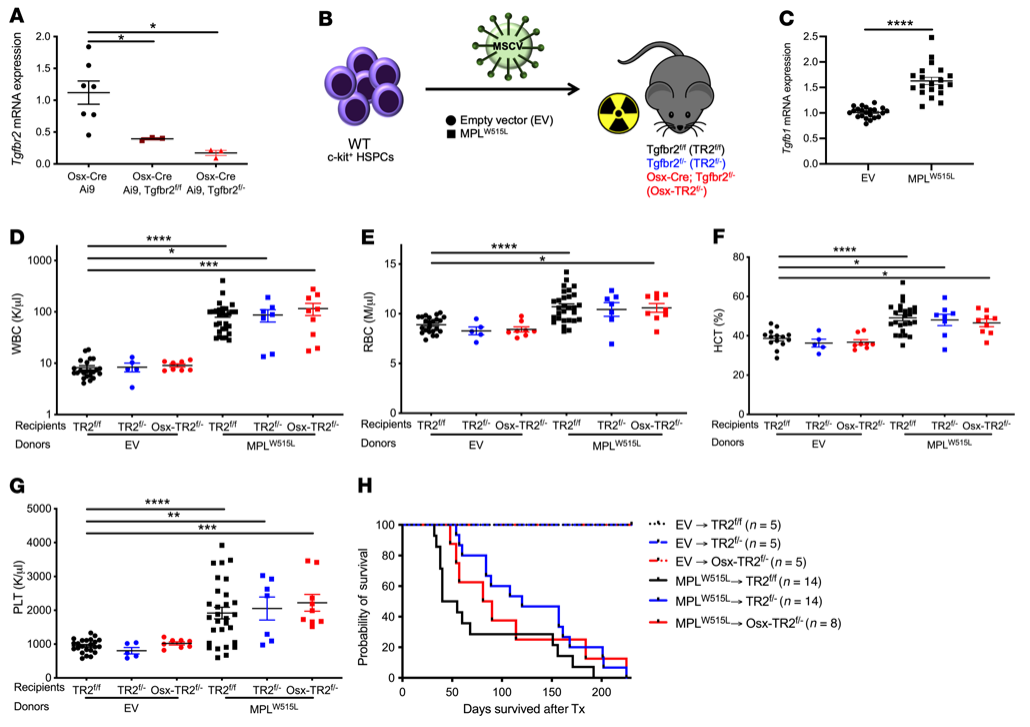
TGF-β signaling in *Osx-Cre*–targeted mesenchymal stromal cells is not required for the development of the myeloproliferative phenotype by *MPL^W515L^*. (**A**) *Tgfbr2* mRNA expression in sorted lineage^–^tdTomato^+^ bone marrow MSCs relative to β-actin mRNA. (**B**) Schematic of experimental design. (**C**) *Tgfb1* mRNA expression relative to β-actin in total bone marrow. (**D**) White blood cell (WBC) count, (**E**) red blood cell (RBC) count, (**F**) hematocrit (HCT), and (**G**) platelet (PLT) count 4–8 weeks after transplantation and (**H**) Kaplan-Meier estimates of survival. EV, empty vector; Tx, transplant. Data presented as the mean ± SEM. *P < 0.05; **P < 0.01; ***P < 0.001; ****P < 0.0001 by 1-way ANOVA (**A** and **D–G**) or Student’s *t* test (**C**).

Prior single-cell RNA sequencing studies of murine bone marrow MSCs show that *Tgfbr2* is broadly expressed in MSCs, including Lepr^+^ perivascular stromal cells and osteolineage cells (27–31). To investigate the contribution of TGF-β signaling in MSCs to the pathogenesis of MPN, we used *Osx-Cre Tgfbr2^fl/fl^* mice as transplant recipients of hematopoietic cells carrying *MPL^W515L^* or *Jak2^V617F^*. The doxycycline-regulated *Osx-Cre* transgene has been shown to target osteoblasts, osteocytes, adipocytes, and perivascular stromal cells (32). Of note, our group recently showed that basal hematopoiesis is normal in *Osx-Cre Tgfbr2^fl/fl^* mice in which expression of Cre recombinase was activated at birth by removal of doxycycline, suggesting that TGF-β signaling in MSCs is dispensable for maintenance of the hematopoietic niche under basal conditions (33). To confirm efficient deletion of *Tgfbr2*, we generated *Osx-Cre Tgfbr2^fl/fl^* Ai9 mice, in which stromal cells targeted by *Osx-Cre* express TdTomato. Although *Tgfbr2* mRNA expression in sorted TdTomato^+^ bone marrow MSCs was significantly reduced in *Osx-Cre Tgfbr2^fl/fl^* mice, Cre-mediated excision was incomplete, with *Tgfbr2* mRNA levels reduced by approximately 66% compared with control cells (Figure 1A). Thus, to increase the percentage of *Tgfbr2*-deleted stromal cells, we generated *Osx-Cre Tgfbr2^fl/–^* Ai9 mice, in which 1 allele of *Tgfbr2* is constitutively deleted. *Tgfbr2* mRNA in TdTomato^+^ stromal cells was reduced to less than 10% of control cells in these mice. Moreover, single-cell RNA sequencing of sorted lineage^–^ (CD45^–^CD3^–^B220^–^Gr-1^–^CD11b^–^Ter119^–^), PDGF receptor β^+^ (PDGFRβ^+^) cells from *Osx-Cre* Ai9 mice confirmed that the majority (>90%) of CXCL12^+^Lepr^+^ stromal cells expressed tdTomato (Supplemental Figure 1; supplemental material available online with this article; https://doi.org/10.1172/JCI154092DS1).

We first used the *MPL^W515L^* retroviral model of MPN in which c-Kit^+^ cells from WT mice transduced with retrovirus expressing *MPL^W515L^* and GFP are transplanted into congenic *Tgfbr2^fl/fl^* or *Osx-Cre Tgfbr2^fl/–^* mice; we also included *Tgfbr2^fl/–^* mice to control for global *Tgfbr2* haploinsufficiency (Figure 1B). Of note, *Tgfb1* mRNA expression in total bone marrow was modestly but significantly elevated in this model (Figure 1C), with an increase in TGF-β^+^ megakaryocytes (Supplemental Figure 2). As reported previously, transplantation of *MPL^W515L^* HSPCs into *Tgfbr2^fl/fl^* recipients resulted in a fatal MPN characterized by leukocytosis and modest erythrocytosis and thrombocytosis (Figure 1, D–H). A similar hematopoietic phenotype was observed in *Osx-Cre Tgfbr2^fl/–^* recipients and animal survival was not altered. These data suggest that TGF-β signaling in MSCs is not required for the development of the lethal myeloproliferative hematopoietic phenotype induced by *MPL^W515L^*.

**Figure 2:**
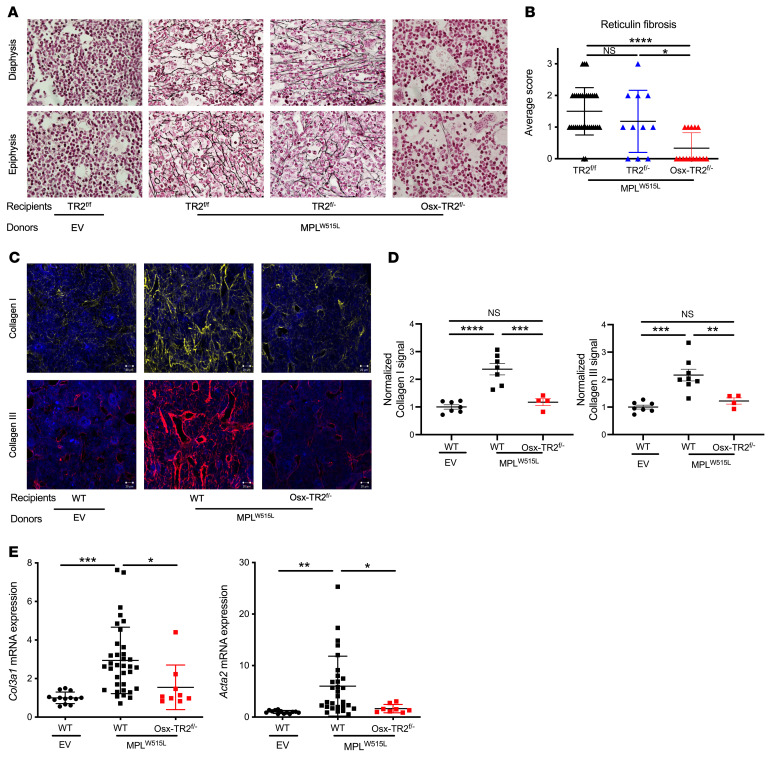
TGF-β signaling in *Osx-Cre*–targeted mesenchymal stromal cells is essential for the development of myelofibrosis by *MPL^W515L^*. (**A**) Representative photomicrographs of femur sections stained for reticulin (original magnification, ×60). (**B**) Average score of fibrosis grading in the diaphysis and epiphysis. (**C**) Representative photomicrographs of femur sections stained for collagen I (yellow) or collagen III (red); nuclei were stained blue with DAPI. Scale bar: 20 μm. (**D**) Normalized fluorescence intensity for collagen I and III. (**E**) mRNA expression levels of collagen 3 (*Col3a1*) and α-smooth muscle actin (*Acta2*) relative to β-actin mRNA in total bone marrow. EV, empty vector; *TR2^fl/fl^*, *Tgfbr2^fl/fl^*; *TR2^fl/–^*, *Tgfbr2^fl/–^*; *Osx-TR2^fl/–^*, *Osx-Cre Tgfbr2^fl/–^*. Mice were analyzed 4–8 weeks after transplantation. Data presented as the mean ± SEM. *P < 0.05; **P < 0.01; ***P < 0.001; ****P < 0.0001 by Kruskal-Wallis test (**B**) or 1-way ANOVA (**D** and **E**).

We next investigated the myelofibrosis phenotype induced by *MPL^W515L^*. As reported previously, transplantation of *MPL^W515L^*-transduced HSPCs induced a moderate to severe reticulin fibrosis in the bone marrow of both *Tgfbr2^fl/fl^* and *Tgfbr2^fl/–^* recipients (Figure 2, A and B). In contrast, reticulin fibrosis was significantly abrogated in *Osx-Cre* *Tgfbr2^fl/–^* recipients. Since we observed that *Tgfbr2^fl/fl^* and *Tgfbr2^fl/–^* recipients have similar phenotypes, we subsequently pooled data from these 2 groups as WT controls. To further characterize the myelofibrosis phenotype, we assessed collagen I and III deposition in the bone marrow. In WT recipients transplanted with *MPL^W515L^*-transduced HSPCs, a marked increase in perivascular collagen I and III was observed, which was abrogated in *Osx-Cre* *Tgfbr2^fl/–^* recipients (Figure 2, C and D). Increased α-smooth muscle actin (*Acta2*) expression has been reported in myelofibrosis (10). Indeed, in the *MPL^W515L^* transplantation model, *Acta2* and *Col3a1* (collagen III) mRNAs were modestly increased, whereas no elevation was observed in *Osx-Cre* *Tgfbr2^fl/–^* recipients (Figure 2E). Interestingly, modest splenic reticulin fibrosis was induced after transplantation of *MPL^W515L^*-transduced HSPCs in both WT and *Osx-Cre* *Tgfbr2^fl/–^* recipients (Supplemental Figure 3). Of note, micro-CT analysis showed that osteosclerosis did not develop in any of the cohorts (Supplemental Figure 4).

**Figure 3:**
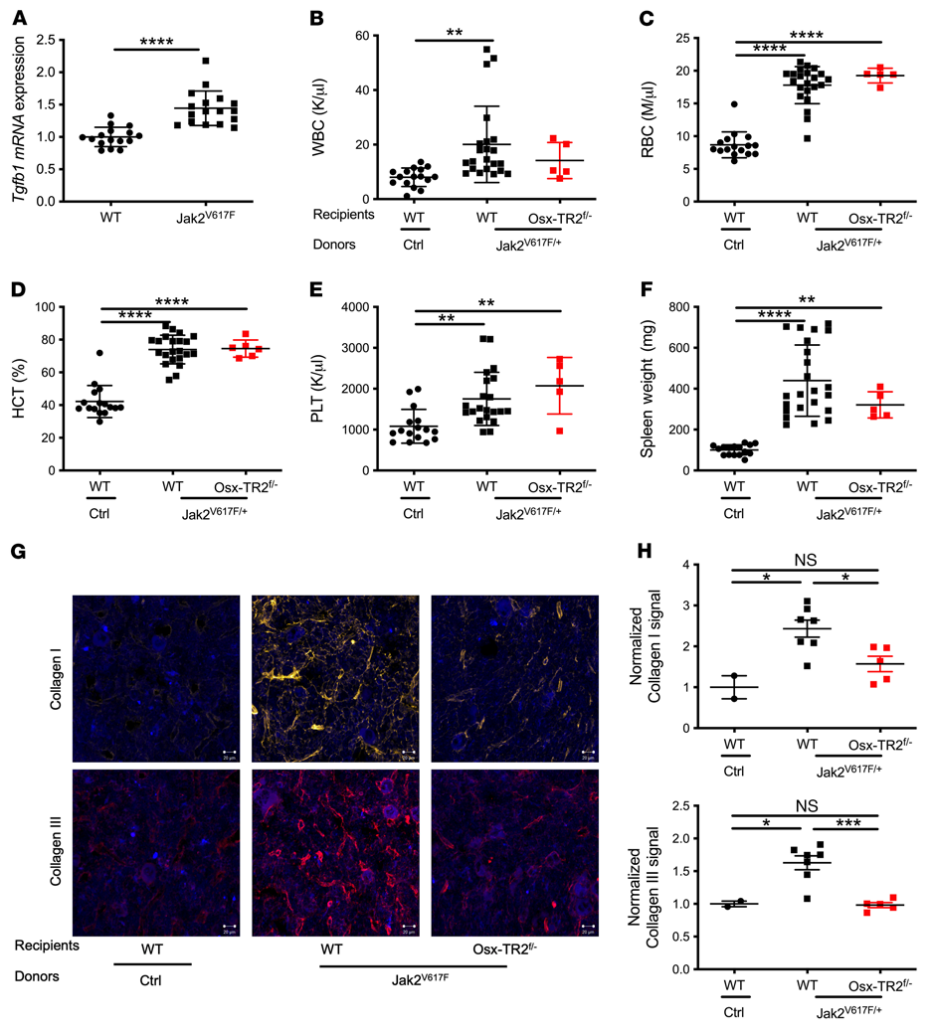
TGF-β signaling in *Osx-Cre*–targeted mesenchymal stromal cells is essential for the development of myelofibrosis by *Jak2^V617F^*. (**A**) *Tgfb1* mRNA expression relative to β-actin mRNA in total bone marrow. (**B**) White blood cell (WBC) count, (**C**) red blood cell (RBC) count, (**D**) hematocrit (HCT), and (**E**) platelet (PLT) count and (**F**) spleen weight 5 months after tamoxifen-induced *Jak2^V617F^* expression. (**G**) Representative photomicrographs of femur sections stained for collagen I (yellow) or collagen III (red); nuclei were stained blue with DAPI. Scale bar: 20 μm. (**H**) Normalized fluorescence intensity for collagen I and III. *Osx-TR2^fl/–^*, *Osx-Cre Tgfbr2^fl/–^*. Data presented as the mean ± SEM. *P < 0.05; **P < 0.01; ***P < 0.001; ****P < 0.0001 by Student’s *t* test (**A**) or 1-way ANOVA (**B–E** and **G**).

Similar results were observed in a *Jak2^V617F^* transgenic model of MPN. Bone marrow from *Jak2^V617F^* mice was transplanted into *Osx-Cre Tgfbr2^fl/–^* or control recipients. Increased *Tgfb1* mRNA expression and TGF-β^+^ megakaryocytes were observed (Figure 3A and Supplemental Figure 2). The hematopoietic phenotype was similar in all mice, including erythrocytosis, leukocytosis, thrombocytosis, and splenomegaly (Figure 3, B–F). As reported previously (34), the degree of myelofibrosis in *Jak2^V617F^* mice was less severe than that observed in the *MPL^W515L^* model, with no consistent reticulin fibrosis seen. However, increased collagen I and III deposition in the bone marrow was observed in control, but not *Osx-Cre Tgfbr2^fl/–^*, recipients (Figure 3, G and H). Altogether, these data show that TGF-β signaling in MSCs is required for the development of myelofibrosis induced by both *MPL^W515L^* and *Jak2^V617F^*.

### Abrogation of TGF-β signaling in MSCs does not rescue the defective hematopoietic niche in MPL^W515L^ mice.

**Figure 4:**
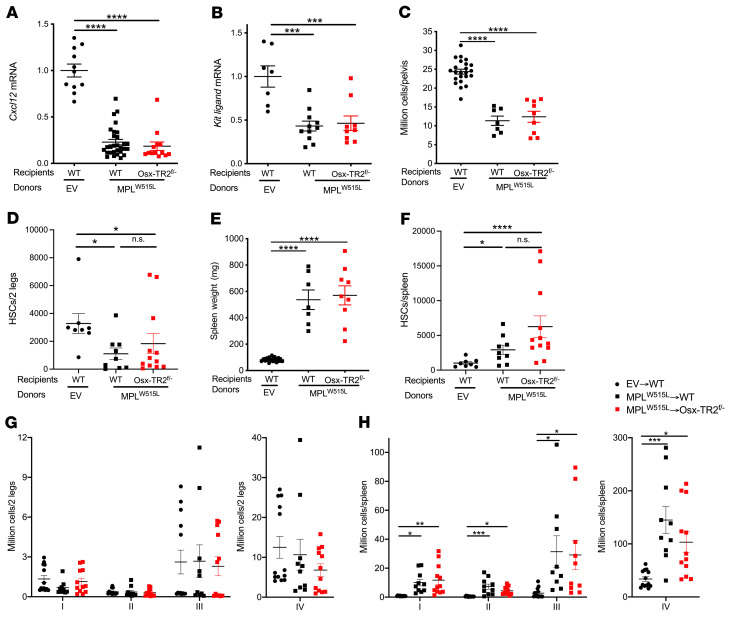
TGF-β signaling in *Osx-Cre*–targeted mesenchymal stromal cells is not required for suppression of niche factor gene expression or development of extramedullary hematopoiesis by *MPL^W515L^*. (**A** and **B**) mRNA expression of *Cxcl12* (**A**) and *Kitlg* (**B**) in total bone marrow relative to β-actin mRNA. (**C**) Bone marrow cellularity per pelvis and (**D**) number of phenotypic HSCs (lineage^–^Sca1^+^c-kit^+^CD150^+^CD48^–^) in the bone marrow. (**E**) Spleen weight and (**F**) HSC number in spleen. (**G** and **H**) Number of erythroid progenitors in bone marrow (**G**) and spleen (**H**). I, proerythroblast; II, basophilic erythroblasts; III, polychromatic erythroblasts; IV, orthochromatic erythroblasts; EV, empty vector; *Osx-TR2^fl/–^*, *Osx-Cre Tgfbr2^fl/–^*. Data presented as the mean ± SEM. *P < 0.05; **P < 0.01; ***P < 0.001; ****P < 0.0001 by 1-way ANOVA.

Decreased stromal cell expression of key niche factors, *Cxcl12* and *Kitlg*, has been reported in several mouse models of MPNs (9–11). Consistent with these reports, we show that *Cxcl12* mRNA expression in the bone marrow of control recipients transplanted with *MPL^W515L^*-transduced HSPCs was markedly decreased (Figure 4A). Surprisingly, a similar decrease in *Cxcl12* mRNA expression was observed in *Osx-Cre Tgfbr2^fl/–^* recipients. Likewise, a modest but significant decrease in *Kitlg* mRNA expression in both control and *Osx-Cre Tgfbr2^fl/–^* recipients was observed (Figure 4B). Of note, we and others previously reported that loss of *Cxcl12* or *Kitlg* expression in bone marrow MSCs is sufficient to disrupt hematopoietic niches, resulting in a decrease in bone marrow cellularity and HSC number and development of extramedullary hematopoiesis (1–3). Indeed, similar decreases in bone marrow cellularity and HSC number were observed in control and *Osx-Cre Tgfbr2^fl/–^* recipients of *MPL^W515L^*-transduced HSPCs (Figure 4, C and D). Moreover, splenomegaly and significant increases in HSCs and erythroid progenitors in the spleen were observed in both control and *Osx-Cre Tgfbr2^fl/–^* recipients (Figure 4, E–H, and Supplemental Figure 5). Collectively, these data suggest that, despite the absence of myelofibrosis, loss of TGF-β signaling in MSCs does not rescue the defective hematopoietic niche induced by *MPL^W515L^*.

### Deletion of Tgfbr2 in osteolineage cells does not affect MPL^W515L^-induced myelofibrosis.

*Osx-Cre* targets a broad range of MSCs, including osteolineage cells, perivascular CXCL12-abundent reticular (CAR) cells, and adipocytes (32). To further identify the stromal cell population responsible for myelofibrosis, we used a *Dmp1-Cre* transgene that targets osteoblasts and approximately 30% of CAR cells, which we previously showed are enriched for osteolineage genes (35). Efficient deletion of *Tgfbr2* was confirmed in TdTomato^+^ stromal cells isolated from *Dmp1-Cre Tgfbr2^fl/fl^* Ai9 mice (Supplemental Figure 6A). As expected, similar alterations in peripheral blood counts, splenomegaly, and bone marrow cellularity were observed in control and *Dmp1-Cre Tgfbr2^fl/fl^* recipients of *MPL^W515L^*-transduced HSPCs (Supplemental Figure 6, B–F). Interestingly, a similar degree of myelofibrosis, as measured by reticulin staining and *Col3a1* mRNA expression, was observed in control and *Dmp1-Cre Tgfbr2^fl/fl^* recipients (Supplemental Figure 6, G–I). These data show that TGF-β signaling in osteolineage cells is not required for the development of myelofibrosis.

### Canonical (SMAD4-dependent) TGF-β signaling in MSCs is not required for MPL^W515L^-induced myelofibrosis.

**Figure 5:**
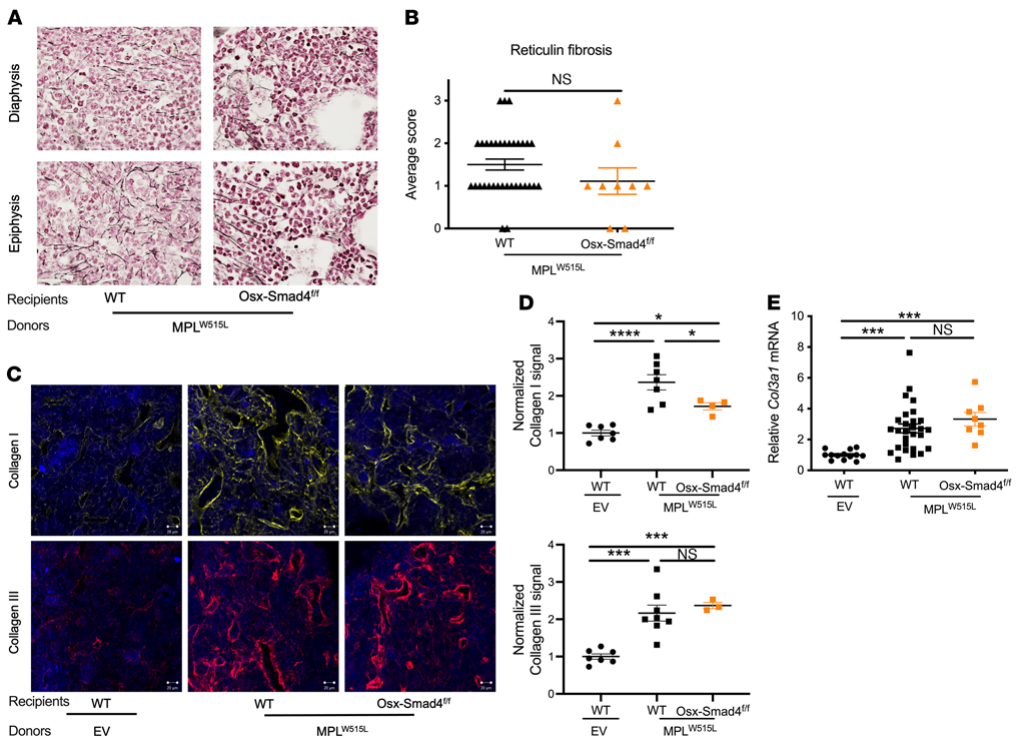
Canonical (SMAD4-dependent) TGF-β signaling in mesenchymal stromal cells is not required for the induction of myelofibrosis by *MPL^W515L^*. (**A**) Representative photomicrographs of femur sections stained for reticulin (original magnification, ×60). (**B**) Average score of fibrosis grading in the diaphysis and epiphysis. (**C**) Representative photomicrographs of femur sections stained for collagen I (yellow) or collagen III (red); nuclei were stained blue with DAPI. Scale bar: 20 μm. (**D**) Normalized fluorescence intensity for collagen I and III. (**E**) *Col3a1* mRNA expression relative to β-actin mRNA in total bone marrow. EV, empty vector; *Osx-Smad4^fl/fl^*, *Osx-Cre Smad4^fl/fl^*. Mice were analyzed 4 weeks after transplantation. Data presented as the mean ± SEM. *P < 0.05; ***P < 0.001; ****P < 0.0001 by Kruskal-Wallis test (**B**) or 1-way ANOVA (**D** and **E**).

TGF-β signaling consists of SMAD-dependent canonical pathways and the SMAD-independent noncanonical pathways. To assess the role of canonical TGF-β signaling in the development of myelofibrosis, we generated *Osx-Cre* *Smad4^fl/fl^* mice. Cre-mediated recombination was induced postnatally by removing doxycycline chow at birth. We previously showed that deletion of *Smad4* is very efficient in *Osx-Cre*–targeted cells, with essentially undetectable *Smad4* mRNA (36). Of note, we reported that these mice have normal basal hematopoiesis, with no identifiable alteration in bone marrow MSCs (36). *MPL^W515L^*-transduced HSPCs were transplanted into *Osx-Cre Smad4^fl/fl^* recipients and similar degrees of leukocytosis, erythrocytosis, thrombocytosis, splenomegaly, and reduction in bone marrow cellularity were observed in control and *MPL^W515L^*-transduced HSPCs (Supplemental Figure 7). Surprisingly, the degree of myelofibrosis was similar in control and *Osx-Cre Smad4^fl/fl^* recipients, as measured by reticulin staining (Figure 5, A and B), collagen I and III immunofluorescence (Figure 5, C and D), and *Col3a1* mRNA expression (Figure 5E). These data suggest that canonical (SMAD4-dependent) TGF-β1 signaling is not required for the development of myelofibrosis in the *MPL^W515L^* murine model of MPN.

### JNK activation by TGF-β contributes to the development of MPL^W515L^-induced myelofibrosis in mice.

**Figure 6:**
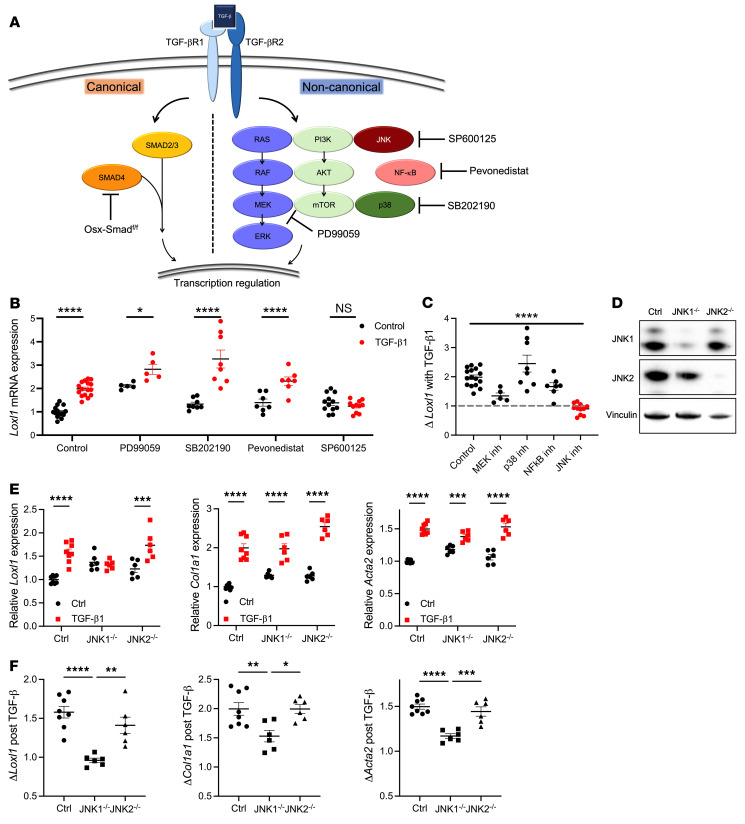
TGF-β contributes to fibrosis-related gene expression through noncanonical JNK signaling. (**A**) TGF-β signaling pathways, with inhibitors used in this study included. (**B**) Relative *Loxl1* mRNA in WT MSC cultures treated with TGF-β1 (10 ng/mL) and one of the following inhibitors: the MEK inhibitor PD99059 (20 μM), the p38 inhibitor SB202190 (20 μM), the NF-κB inhibitor pevonedistat (1 μM), or the JNK inhibitor SP600125 (20 μM). (**C**) Fold change (Δ) in *Loxl1* mRNA expression induced by TGF-β1 in the presence of the indicated inhibitors. (**D**) Immunoblot showing JNK1 and JNK2 protein expression in control or *Mapk8*-deleted (JNK1^–/–^) or *Mapk9*-deleted (JNK2^–/–^) cells. (**E**) Relative *Loxl1*, *Col1a1*, and *Acta2* mRNA in MSCs treated with TGF-β1 (10 ng/mL). (**F**) Fold change in *Loxl1*, *Col1a1*, and *Acta2* mRNA expression induced by TGF-β1. Data presented as the mean ± SEM. *P < 0.05; **P < 0.01; ***P < 0.001; ****P < 0.0001 by 2-way ANOVA (**B** and **E**) or 1-way ANOVA (**C** and **F**).

Noncanonical TGF-β signaling includes the activation of extracellular signal–regulated kinase (ERK), JNK, p38, PI3 kinase, and NF-κB pathways (Figure 6A and ref. 37). Treatment of cultured bone marrow MSCs with TGF-β1 resulted in increased expression of several fibrosis-associated genes, including *Loxl1* (which encodes lysyl oxidase–like 1), *Col1a1*, and *Acta2* (Figure 6B and Supplemental Figure 8, A–D). It also resulted in Smad2 and c-Jun phosphorylation, but not ERK1/2 phosphorylation (Supplemental Figure 8E). Consistent with this result, treatment with PD99059, a MEK inhibitor, resulted in a modest change in the basal expression of these genes, but it did not block their induction by TGF-β1 (Figure 6, B and C, and Supplemental Figure 8, A–D). Treatment with SB202190 (a p38 inhibitor) or pevonedistat (an NF-κB inhibitor) was associated with a significant decrease in basal *Col1a1* and *Acta2* mRNA expression, but also did not prevent TGF-β1–induced increases in the expression of these genes (Figure 6, B and C, and Supplemental Figure 8, A–D). In contrast, treatment with SP600125, a pan-JNK inhibitor, significantly reduced TGF-β1–induced increases in *Loxl1*, *Col1a1*, and *Acta2* mRNA expression (Figure 6, B and C, and Supplemental Figure 8, A–D).

JNK1 and JNK2, but not JNK3, are expressed in bone marrow MSCs (38). To determine whether JNK1 or JNK2 selectively contributes to TGF-β–induced myelofibrosis, we used CRISPR/Cas9 gene editing to delete *Mapk8* (JNK1) or *Mapk9* (JNK2) in MSCs. Both JNK1 and JNK2 were efficiently deleted (Figure 6D). Deletion of JNK1 completely abrogated TGF-β–induced *Loxl1* expression and significantly reduced TGF-β–induced *Col1a1* and *Acta2* expression (Figure 6, E and F). However, deletion of JNK2 did not affect TGF-β–induced expression of fibrosis-related genes. Collectively, these data suggest that TGF-β1–induced activation of JNK1 is the major signal mediating myelofibrosis.

**Figure 7:**
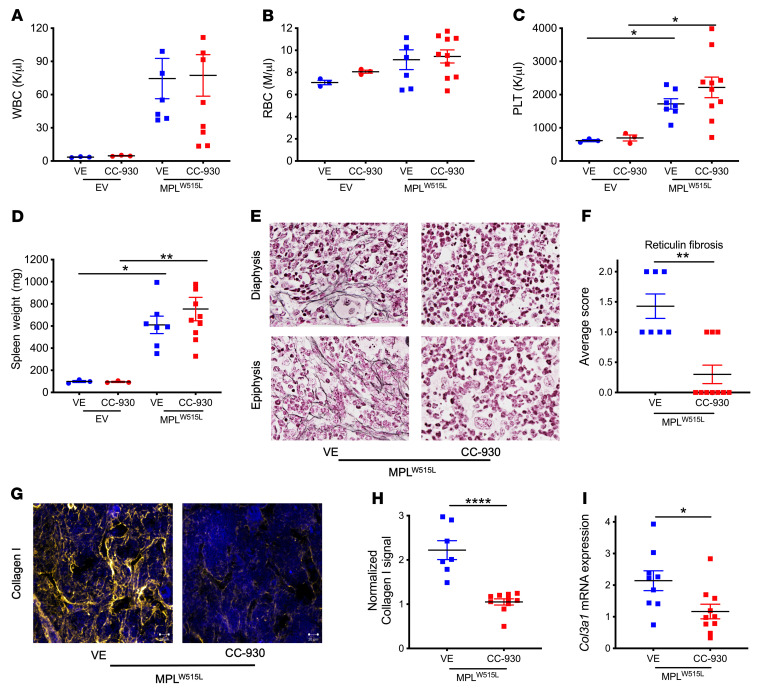
JNK inhibitor alleviates fibrosis phenotypes in *MPL^W515L^*-induced MPN. WT mice transplanted with *MPL^W515L^*-transduced HSPCs were treated with pan-JNK inhibitor CC-930 (tanzisertib) twice daily for 4 weeks beginning 2 weeks after transplantation. (**A**) White blood cell (WBC) count, (**B**) red blood cell (RBC) count, (**C**) platelet (PLT) count, and (**D**) spleen weight. (**E**) Representative photomicrographs of femur sections stained for reticulin (original magnification, ×60). (**F**) Average score of fibrosis grading in the diaphysis and epiphysis. (**G**) Representative photomicrographs of femur sections stained for collagen I (yellow) or collagen III (red); nuclei were stained blue with DAPI. Sacle bar: 20 μm. (**H**) Normalized fluorescence intensity for collagen I. (**I**) *Col3a1* mRNA expression relative to β-actin mRNA in total bone marrow. EV, empty vector; VE, vehicle-treated control. Data presented as the mean ± SEM. *P < 0.05, **P < 0.01, ****P < 0.0001 by 1-way ANOVA (**A**–**D**), Kruskal-Wallis test (**F**), or Student’s *t* test (**H** and **I**).

Finally, to assess the impact of JNK activation on the development of myelofibrosis, we treated WT mice transplanted with *MPL^W515L^*-transduced HSPCs with the pan-JNK inhibitor CC-930 (tanzisertib) twice daily for 4 weeks beginning 2 weeks after transplantation. Of note, this regimen was effective in blocking JNK signaling in lineage^–^ bone marrow MSCs (Supplemental Figure 9). Treatment with CC-930 had no significant effect on *MPL^W515L^*-induced leukocytosis, erythrocytosis, thrombocytosis, or splenomegaly (Figure 7, A–D). However, treatment with CC-930 resulted in the nearly complete abrogation of myelofibrosis, as assessed by reticulin fibrosis, collagen I deposition, and *Col3a1* mRNA expression (Figure 7, E–I). These data show that treatment with the pan-JNK inhibitor CC-930 prevents induction of myelofibrosis by *MPL^W515L^* in mice.

## Discussion

In this study, we provide strong evidence that TGF-β signaling in *Osx-Cre*–targeted MSCs plays an essential role in the develop of myelofibrosis. These results are consistent with prior studies showing that genetic or pharmacologic inhibition of TGF-β signaling reduces reticulin fibrosis in *TPO* overexpression (20) or *GATA1^low^* (21) models of MPNs. In addition, Yue et al. showed that inhibition of TGF-β using galunisertib, an ALK5 (TGFBR1) inhibitor, reduces, but does not abrogate, myelofibrosis in the clinically relevant *MPL^W515L^* model of MPN. Of note, TGF-β signals through heterodimers of TGFBR2 with either TGFBR1 (ALK5) or ACVRL1 (ALK1), both of which are expressed at similar levels in bone marrow MSCs (28). Thus, the residual myelofibrosis in galunisertib-treated mice may be due to ALK1-dependent TGF-β signaling. Consistent with this conclusion, we show that genetic abrogation of all TGF-β signaling (by deleting *Tgfbr2*) in MSCs completely prevented the development of myelofibrosis in the majority of mice in both *MPL^W515L^* and *Jak2^V617F^* models of MPNs. Although requiring experimental validation, we suspect that the low-grade myelofibrosis observed in a minority of *Osx-Cre Tgfbr2^fl/–^* recipients is due to inefficient Cre-mediated excision of *Tgfbr2*.

Prior studies have implicated other inflammatory mediators in the pathogenesis of myelofibrosis. Decker et al. showed that reduction of PDGF signaling in MSCs by deletion of *Pdgfra* or by treatment with imatinib reduced myelofibrosis in the *TPO* overexpression model of MPN (9). However, treatment with PDGF did not induce fibrogenic gene expression in cultured bone marrow MSCs (Supplemental Figure 10). Whether PDGF signaling in MSCs provides a permissive signal required for TGF-β–induced fibrogenic gene expression will require further study. Schenider et al. reported that increased hematopoietic cell expression of *Cxcl4* contributes to the development of myelofibrosis in the *TPO* overexpression MPN model (10, 23). However, the known receptors for CXCL4, CXCR3, and CCR1 are not expressed on bone marrow MSCs (28), suggesting that CXCL4 acts in an indirect fashion to induce myelofibrosis. Consistent with this conclusion, Schneider et al. showed that *TPO*-induced expression of TGF-β in *Cxcl4^–/–^* megakaryocytes was attenuated (23). Thus, CXCL4 may act upstream of TGF-β to contribute to myelofibrosis development.

Our data suggest that perivascular MSCs, but not osteoblast-lineage cells, are the key drivers of myelofibrosis. Specifically, we show that abrogation of TGF-β signaling in *Osx-Cre*–targeted, but not *Dmp1-Cre*–targeted stromal cells, prevents the development of myelofibrosis. These results are consistent with recent studies suggesting that Lepr^+^ or Gli1^+^ MSCs in the bone marrow are the key drivers of myelofibrosis (9, 10). Of note, constitutively active *Lepr-Cre*, similarly to postnatal active *Osx-Cre*, targets the majority of perivascular MSCs and osteoblasts (39, 40). Likewise, *Gli1-Cre* targets both perivascular and endosteal MSCs, including osteoblasts (41). Leimkuhler et al. recently reported results of single-cell RNA sequencing of bone marrow MSCs in a *TPO* overexpression model of MPN (11). They showed that adipogenic and osteogenic primitive subsets of Lepr^+^ MSCs expanded and upregulated expression of fibrosis-related genes. Of note, we previously showed that *Dmp1-Cre* targets an osteogenic subset of CAR (or Lepr^+^) stromal cells (35). Together, these data suggest that the adipogenic subset of Lepr^+^ MSCs is the cell of origin for myelofibrosis.

Our data show that noncanonical JNK-dependent TGF-β signaling is responsible for the induction of myelofibrosis. Deletion of *Smad4* in bone marrow MSCs does not prevent the induction of myelofibrosis by *MPL^W515L^*. In contrast, treatment with a pan-JNK inhibitor abrogated the development of myelofibrosis by *MPL^W515L^*. This observation is consistent with a prior study showing that expression of c-Jun, a downstream target of JNK, is increased in all major human fibrotic conditions, including myelofibrosis (42). Another prior report also found activation of noncanonical TGF-β signaling, including JUN, in patients with myelofibrosis (43). Of note, there is evidence of cross-talk between canonical and noncanonical TGF-β signaling. Of particular relevance are data showing that JNK can phosphorylate and activate SMAD3 (44, 45). Whether the loss of SMAD3 activity contributes to suppression of myelofibrosis after JNK inhibitor treatment will require further study.

Our data suggest that treatment with JNK inhibitors may have therapeutic benefit in patients with myelofibrosis, although there are several important caveats. First, it is not clear whether JNK inhibitors are able to reverse myelofibrosis after it is established. Second, our data suggest that reversal of myelofibrosis may not improve hematopoietic niche function, raising questions about whether this treatment would significantly impact cytopenias present in many patients with MPN. Finally, a clinical trial of CC-930 (the pan-JNK inhibitor used in our study) in patients with idiopathic pulmonary fibrosis (NCT01203943) was terminated due to an unfavorable benefit/risk profile. Of note, preclinical studies suggesting a selective role for JNK1 in pulmonary fibrosis have led to an ongoing clinical trial of CC-90001, a JNK1-selective inhibitor, in patients with idiopathic pulmonary fibrosis (NCT03142191; ref. 46). Likewise, our in vitro data suggest that JNK1 is the major mediator of fibrosis-related gene expression in mouse bone marrow MSCs. Whether treatment with a JNK1-selective inhibitor would prevent or attenuate myelofibrosis will require further study.

In contrast to myelofibrosis, our data show that TGF-β signaling in MSCs is not required for the disruption of hematopoietic niches in MPNs. Specifically, the loss of TGF-β signaling in MSCs did not prevent the suppression of key niche factors, *Cxcl12* and *Kitlg*, nor did it prevent the development of extramedullary hematopoiesis or loss of bone marrow HSCs. Prior studies have established the importance of *Cxcl12* and *Kitlg* expression in MSCs in the maintenance of a functioning hematopoietic niche. Indeed, deletion of these genes in *Osx-Cre*–targeted (or *Lepr-Cre*–targeted) stromal cells results in HSPC mobilization and development of extramedullary hematopoiesis (1–3). Based on these observations, we propose that suppression of MSC *Cxcl12* and/or *Kitlg* expression plays a key role in the disruption of the hematopoietic niche and development of extramedullary hematopoiesis, although we acknowledge that additional data from patients with MPNs is needed to confirm. These data also show for the first time to our knowledge that the signals that induce a fibrogenic program in bone marrow MSCs are distinct from those that suppress *Cxcl12* and *Kitlg* expression. Our data show that the fibrogenic program is dependent on TGF-β signaling, while the signals that regulate niche factor expression remain unknown.

In summary, in this study, we show that noncanonical JNK-dependent TGF-β signaling in perivascular MSCs is responsible for the development of myelofibrosis but not hematopoietic niche disruption. JNK is a druggable target that may have therapeutic benefit in the treatment and/or prevention of myelofibrosis in patients with MPNs.

## Methods

### Mice.

*Tgfbr2^fl/fl^* (B6;129-*Tgfbr2^tm1Karl^*/J; ref. 47), *Osx-Cre* (B6.Cg-Tg ^Sp7-tTA,tetO-EGFP/cre^1Amc/J; ref. 48), Ai9 [B6.Cg-*Gt(ROSA)26Sor^tm9(CAG-tdTomato)Hze^*/J; ref. 49], *UBC-Cre^ERT2^* (B6.Cg-*Ndor1^Tg(UBC-cre/ERT2)1Ejb^*/1J; ref. 50), *Jak2^V617F^* [B6N.129S6(SJL)-*Jak2^tm1.2Ble^*/AmlyJ; ref. 34], and *Dmp1-Cre* [B6N.FVB-Tg(Dmp1-cre)1Jqfe/BwdJ; ref. 51] mice were obtained from The Jackson Laboratory. *Tgfbr2^fl/–^* mice were generated by breeding *Tgfbr2^fl/fl^* with *E2A-Cre* [B6.FVB-Tg(EIIa-cre)C5379Lmgd/J; ref. 52] mice. *Smad4^fl/fl^* mice were a gift from Fanxin Long, University of Pennsylvania School of Medicine, Philadelphia, Pennsylvania, USA. All mice were back-crossed onto the C57BL/6 background and were maintained under standard pathogen-free conditions. All mice used in this study were 6 to 12 weeks old. Both male and female mice were used equally in these studies. To induce recombination postnatally, *Osx-Cre* *Tgfbr^fl/–^* or *Osx-Cre Smad4^fl/fl^* breeders were maintained on 200 ppm doxycycline chow, which was removed after pups were born.

### MPL^W515L^ MPN model.

c-Kit^+^ HSPCs from WT mice were enriched using murine CD117 (c-Kit) microbeads and the autoMACS Pro Separator system, per the manufacturer’s instructions (Miltenyi Biotec) and cultured overnight at 37°C with 5% CO_2_ in stem cell media (MEM-α with 10% FBS, 100 U penicillin and streptomycin, 10 ng/mL murine TPO [mTPO], 10 ng/mL mIL-3, 10 ng/mL mFLT3L, and 100 ng/mL mSCF). To generate retrovirus, the retroviral packaging vector pCL-Eco was cotransfected with either MSCV-MPL^W515L^-IRES-GFP or MSCV-IRES-GFP (empty vector) into 293T cells using calcium phosphate precipitation. Freshly generated retrovirus was added to HSPC cultures containing polybrene (6 mg/mL). Cells were spin-infected at 1200*g* at 30°C for 90 minutes and then cultured overnight. Five hundred thousand transduced c-Kit^+^ cells were injected retro-orbitally into recipients, which had been irradiated with two 600-cGy doses, 16 hours apart. Recipient mice were placed on prophylactic antibiotics (trimethoprim-sulfamethoxazole) for 2 weeks following transplantation. Mice were sacrificed for analysis when at least one of the mice in the same transplanted cohort became moribund, which was around 4 to 8 weeks after transplantation. Of note, at time of terminal analysis, more than 95% of hematopoietic cells were GFP^+^.

### Jak2^V617F^ MPN model.

Donor bone marrow cells were collected from 6- to 8-week-old *UBC-Cre^ERT2^ Jak2^V617F^* or *UBC-Cre^ERT2^* mice, and 2 million bone marrow cells were injected retro-orbitally into lethally irradiated recipients. Six weeks after transplantation, mice were treated with 3 mg tamoxifen in corn oil daily for 5 days via oral gavage to induce Cre recombinase activity. Mice were sacrificed for analysis 5 months after tamoxifen induction.

### Immunostaining of femur sections.

Mouse femurs were fixed in 10% formalin for 24 to 48 hours at 4°C. Bones were then decalcified in 14% EDTA, pH 7.4, for 10 to 14 days at 4°C. Following incubation in 30% sucrose for 24 hours at 4°C to dehydrate, bones were embedded in optimal cutting temperature compound (OTC, Sakura Finetek). These tissue blocks were cut into 12-μm sections using a CryoJane system (Leica Biosystems). For immunostaining, the slides were blocked with 5% donkey serum in TBST for 1 hour at room temperature. Slides were then incubated with primary antibody overnight at 4°C, followed by incubation with secondary antibody for 1 hour at room temperature. The following antibodies were used: rabbit anti–collagen I (Abcam, ab34710), rabbit anti–collagen III (Abcam, ab7778), goat anti–TGF-β1 (LAP) (R&D Systems, AB-246-NA), PE–anti-CD41 (BD Biosciences, MWReg30), and Alexa Fluor 647–conjugated donkey anti–rabbit IgG (H+L) (Thermo Fisher Scientific). Finally, slides were mounted with ProLong Gold antifade reagent with DAPI (Life Technologies). Images were acquired with an LSM 700 microscope (Carl Zeiss). Fluorescence intensity was quantified using ImageJ (NIH).

### Femur and spleen histology.

Mouse femurs and spleens were fixed in 10% formalin for 24 hours at 4°C. Femurs were then decalcified in 14% EDTA, pH 7.4, for 10–14 days at 4°C. Femurs and spleens were dehydrated via incubation in 30%, 50%, and 70% ethanol for 15 minutes each. Processed bones and spleens were further embedded in paraffin and sectioned into 5-μm sections and stained for reticulin fibers by the Musculoskeletal Research Histology Core at Washington University.

### Histologic analysis of femur and spleen tissue.

The degree of fibrosis was graded in blinded fashion by 2 independent hematopathologists using adjusted WHO criteria (53). Scores were assigned based on the degree of fibrosis as well as the percentage of the femur that was fibrotic: 0, no fibrosis; 1, mild and patchy fibrosis; 2, moderate and patchy fibrosis; and 3, moderate to severe uniform fibrosis.

### Quantitative real-time PCR.

To collect total bone marrow RNA, tibias from mice were either crushed (*MPL^W515L^* mice) with a mortar and pestle in 1 mL of TRIzol (Invitrogen) or flushed (*Jak2^V617F^* mice) with 1 mL of TRIzol. RNA was extracted following the manufacturer’s instructions. cDNA was prepared using iScript reverse transcription supermix (Bio-Rad). Quantitative real-time PCR (qRT-PCR) was performed using the TaqMan Universal RT Master Mix (Applied Biosystems). Data were collected on a StepOnePlus Real-Time PCR System (Applied Biosystems). TaqMan probe primers were all from Thermo Fisher Scientific: *Actb* VIC-MGB (Mm04394036_g1), *Tgfb1* FAM-MGB (Mm01178820_m1), *Tgfbr2* FAM-MGB (Mm03024091_m1), *Col1a1* FAM-MGB (Mm00801666_g1), *Col3a1* FAM-MGB (Mm01254476_m1), *Acta2* FAM-MGB (Mm01546133_m1), and *Loxl1* FAM-MGB (Mm01145738).

### Flow cytometry.

Bone marrow and spleen were processed for flow cytometry as previously described (54). Cells were analyzed on a Gallios flow cytometer (Beckman Coulter). Data analysis was done using FlowJo version 10.7.1 software (Tree Star). The following antibodies were used for staining murine cells: anti–Gr-1 (clone RB6-8C5), anti-Ter119 (clone TER-119), anti-CD117 (clone 2B8), anti-CD48 (clone HM48-1), and anti-CD140b (PDGFRβ, clone APB5) from eBioscience; anti-CD45 (clone HI30), anti-CD11b (clone M1/70), anti-B220 (clone RA3-6B2), anti-CD3e (clone 145-2C11), anti-CD115 (clone AFS98), anti–Sca-1 (clone D7), anti-CD150 (clone TC15-12F12.2), and anti-CD71 (clone RI7217) from BioLegend.

### Micro-CT analysis.

Mouse femurs were fixed in 10% formalin for 24 hours at 4°C and then dehydrated in 70% ethanol. Processed tissues were scanned at 10-μm voxel resolution using a Scanco μCT 40 by the Musculoskeletal Research Histology Core at Washington University.

### Bone marrow MSC culture.

Total bone marrow cells from 3- to 4-week-old WT or *Osx-Cre* *Smad4^fl/fl^* mice were collected and cultured in MSC media (DMEM, 20% FBS, 100 U penicillin and streptomycin) overnight at 37°C with 5% CO_2_. Nonattached cells were removed the next day by gently aspirating the media. Cells were washed with PBS and fresh medium was added every day for another 2 days. Attached MSCs were expanded for 3 more days until they reached approximately 80% confluence. MSCs strongly attach to cell culture plates, so after trypsinization, cells were manually scraped to ensure efficient collection. For TGF-β1 and PDGF stimulation experiments, 200,000 MSCs were plated per well in 6-well plates and 10 ng/mL TGF-β1, PDGF-AA, PDGF-BB, or vehicle control (1% bovine serum albumin in PBS) was added every 24 hours for 3 days. For MAP kinase inhibitor treatments, 20 μM MEK inhibitor (PD99059), p38 inhibitor (SB202190), or JNK inhibitor (SP600125), or 1 μM NF-κB inhibitor (pevonedistat) or vehicle control (DMSO) were added every 24 hours for 3 days. Twenty-four hours after the last treatment, cells were lysed with TRIzol, and RNA was extracted for quantification using qRT-PCR analysis.

### CRISPR/Cas9 gene editing.

Recombinant Cas9 protein (0.25 μL of 62 μM solution; Alt-R *Streptococcus* *pyogenes* Cas9 Nuclease V3, IDT), 1 μL of sgRNAs (50 μM), and 1 μL Buffer T (Neon, Invitrogen) were incubated at room temperature for 15 to 30 minutes. Cultured MSCs (100,000 cells) were washed with PBS, resuspended in Buffer T at 13,000 cells/μL, and then added to the Cas9/sgRNA mix. Cells were nucleofected with 1 pulse at 1500 V for 20 ms using the Neon transfection system (Invitrogen). Cells were allowed to expand in culture for 72 hours after nucleofection before stimulating with TGF-β1. For each gene, 3 different sgRNAs targeting the same exon were pooled to enhance editing efficiency. Sequences of the sgRNAs targeting exon 3 of *Mapk8* (JNK1) were 5′-CATAAGAACTAGTTCTCGGT-3′, 5′-GGGTCTGATTCTGAAATGGC-3′, and 5′-TCGGTAGGCGCGCTTAGCAT-3′. Sequences of the sgRNAs targeting exon 3 of *Mapk9* (JNK2) were 5′-TAAGAGGACGAGTTCACGGT-3′, 5′-TACAGTTCTTGGGATAAATG-3′, and 5′-AGGCTCTCTTTGCGTGCGTT-3′.

### Immunoblotting.

MSCs were lysed with RIPA buffer (MilliporeSigma) per the manufacturer’s instructions, and proteins were denatured in SDS sample buffer with 9% β-mercaptoethanol at 100°C for 5 minutes. The following antibodies were used (all purchased from Cell Signaling Technology): anti-SMAD2 (catalog 5339), anti–phospho-SMAD2 (catalog 3104), anti–c-JUN (catalog 9165), anti–phospho-c-JUN (catalog 3270), anti-ERK1/2 (catalog 4695), anti–phospho-ERK1/2 (catalog 4370), anti-JNK1 (catalog 3708), anti-JNK2 (catalog 4672), and anti-vinculin (catalog 13901).

### JNK inhibitor treatment in mice.

*MPL^W515L^* retrovirus–transduced HSPCs were transplanted into lethally irradiated WT recipients. Two weeks after transplantation, mice were treated with vehicle control (0.5% carboxymethyl cellulose, 0.25% Tween 80) or 50 mg/kg CC-930 (55) (MedChemExpress) twice daily via oral gavage for 4 weeks.

### Single-cell RNA sequencing.

Mouse bone marrow from 1 male and 1 female *Osx-Cre* Ai9 mouse was crushed and dissociated with collagenase II (Worthington). Cells were sorted for lineage^–^ and PDGFRβ^+^. Sorted cells were captured using 10× Genomics Chromium 3′ gel beads version 3.1. Gene expression libraries were sequenced on a NovaSeq S4 with the goal of achieving 50,000 reads per cell. A modified reference sequence was created to incorporate the Ai9 reporter sequence (positions 3897–6222; https://support.10xgenomics.com/single-cell-gene-expression/software/pipelines/latest/using/count) into mm10 for mapping using Cell Ranger v6. Quality filters were applied to remove cells with mitochondrial transcripts greater than 10% and UMI counts greater than 50,000. Log normalization, PCA, dimensional reduction, and clustering were performed using the Seurat (v4.1.0) package in R (v4.1.1); https://www.r-project.org The single-cell RNA expression data have been submitted to NCBI’s Gene Expression Omnibus (GEO GSE200693).

### Statistics.

Unpaired Student’s *t* test was used to evaluate the significance of differences between 2 groups. One-way ANOVA was used to evaluate the significance of differences between multiple groups. The Kruskal-Wallis test was used to evaluate the significance of differences of the categorical fibrosis grades. All data are presented as mean ± SEM. A *P* value of less than 0.05 was considered significant.

### Study approval.

All animal studies were approved by the Institutional Animal Care and Use Committee at Washington University.

## Authors contributions

JCY and DCL conceived and designed the experiments, analyzed the data, and wrote the manuscript. JCY performed the experiments. KAO performed and analyzed the single-cell RNA sequencing data. GAE generated the *Osx-Cre Tgfbr2^fl/fl^* mice. JRK generated the *Osx-Cre Smad4^fl/fl^* mice. SU performed the MSC culture experiments. EJD, TW, and HX performed the grading of fibrosis and captured images of bone marrow and spleen histology staining. DCL supervised the research.

## Supplementary Material

Supplemental data

## Version 1 04/19/2022

In-Press Preview

## Version 2 06/01/2022

Electronic publication
