# Supplementary material for: TGF-β signaling in myeloproliferative neoplasms contributes to myelofibrosis without disrupting the hematopoietic niche
Source: J Clin Invest. 2022 Jun 1;132(11):e154092. doi: 10.1172/JCI154092 (PMC9151699; doi:10.1172/JCI154092)
Supplement: Supplemental data [file jci-132-154092-s008.pdf]

SUPPLEMENTARY FIGURES

Table of contents:

Supplementary Figure 1. Single cell RNA sequencing of sorted stromal cells

Supplementary Figure 2. Megakaryocytes express TGF-β

Supplementary Figure 3. Reticulin fibrosis in the spleen of *MPL*^{W515L} induced MPN.

Supplementary Figure 4. Micro-CT analysis shows no significant changes in trabecularization in *MPL*^{W515L} induced MPN.

Supplementary Figure 5. Erythroid progenitor analysis.

Supplementary Figure 6. TGF-β signaling in *Dmp1-Cre* targeted osteolineage cells is not required for the induction of myelofibrosis by *MPL*^{W515L}.

Supplementary Figure 7. Canonical (SMAD4-dependent) TGF-β signaling in *Osx-Cre* targeted mesenchymal stromal cells is not required for the development of the myeloproliferative phenotype by *MPL*^{W515L}.

Supplementary Figure 8. *Acta2* and *Loxl1* mRNA expression in cultured MSC treated with kinase inhibitors and TGF-β1.

Supplementary Figure 9. CC-930 blocks JNK signaling activation *in vivo*.

Supplementary Figure 10. *Col1a1* and *Acta2* mRNA expression in cultured MSC treated with TGF-β1 or PDGFs.

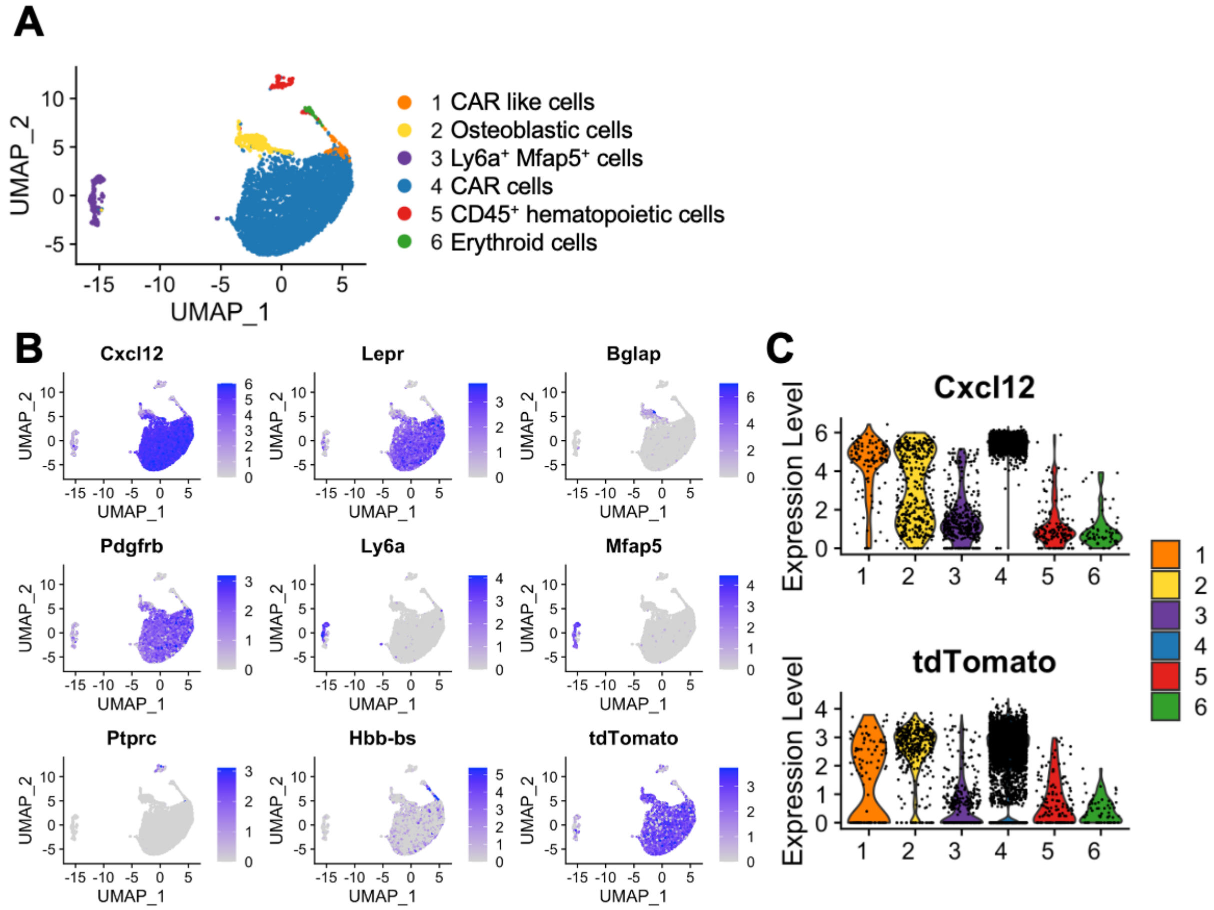

Supplementary Figure 1. Single cell RNA sequencing of sorted stromal cells. (**A**) Annotations of single cell RNA sequencing clusters from sorted lineage^{-} (CD45, CD3, B220, Gr-1, CD11b, Ter119) Pdgfrb^{+} cells. CAR cells: Cxcl12 Abundant Reticular cells. (**B**) Expression of indicated cell type specific markers as well as *tdTomato*. (**C**) Violin plots showing expression *Cxcl12* and *tdTomato* in all clusters.

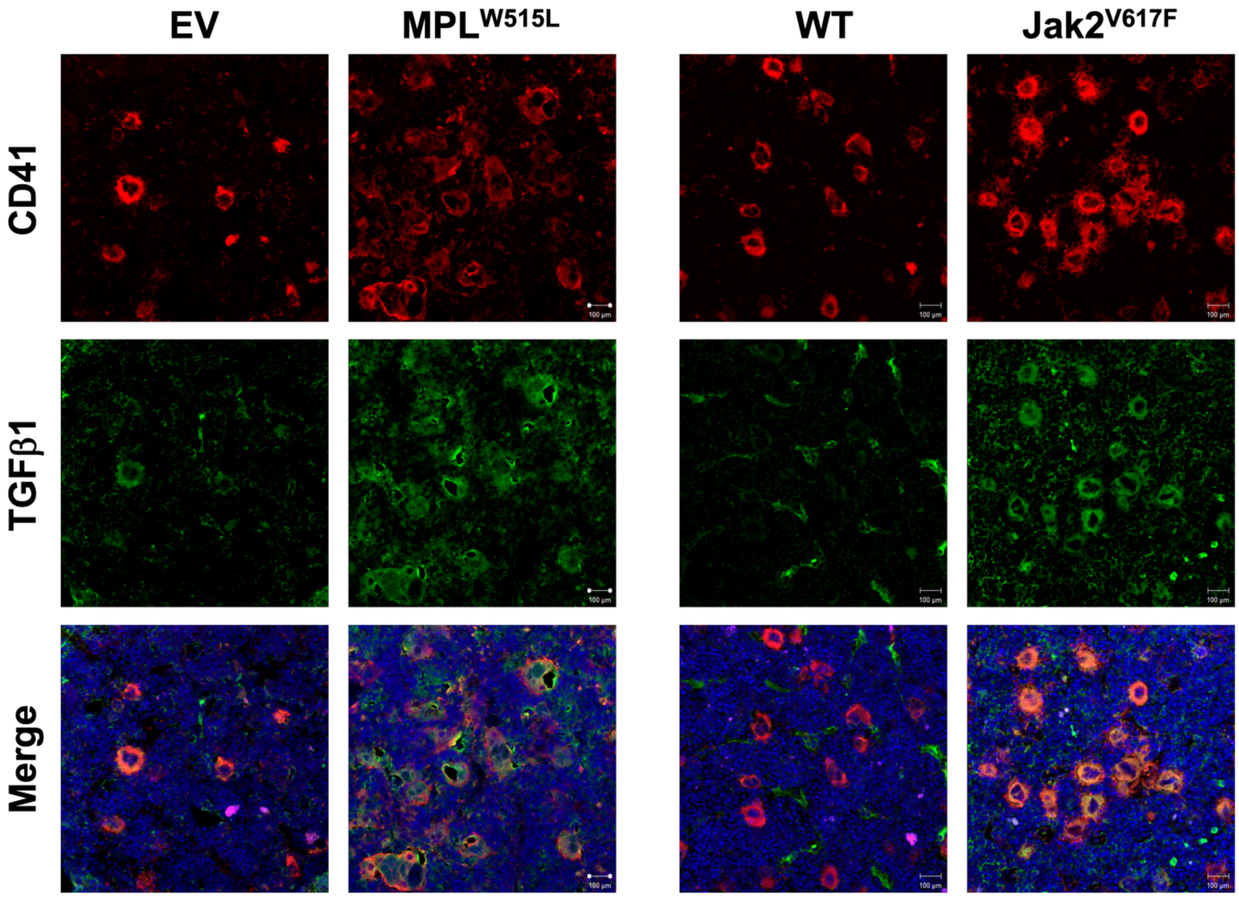

Supplementary Figure 2. Megakaryocytes express TGF-β in MPN mouse models. Immunostaining showing overlapping of megakaryocyte marker CD41 and TGF-β1 in MPL^{W515L} and Jak2^{V617F} mouse models of MPNs. EV: empty vector control. Red: CD41 stains megakaryocytes, green: TGF-β1, blue: DAPI stains nuclei.

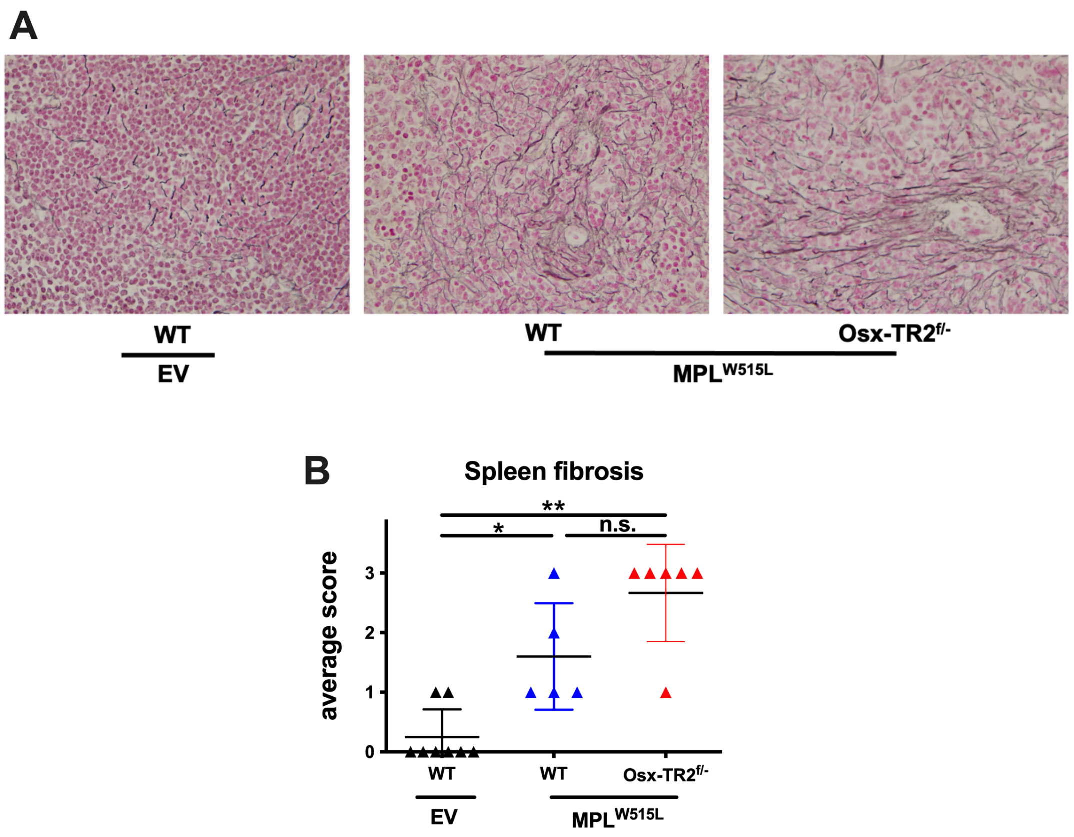

Supplementary Figure 3. Reticulin fibrosis in the spleen of *MPL^{W515L}* induced MPN. (**A**) Representative photomicrographs of spleen sections stained for reticulin (20X). (**B**) Average score of fibrosis grading in the spleen. Osx-TR2$^{f/-}$: Osx-Cre; Tgfbr2$^{f/-}$; EV: empty vector. Data represent the mean ± SEM. Significance calculated by Mann-Whitney test.

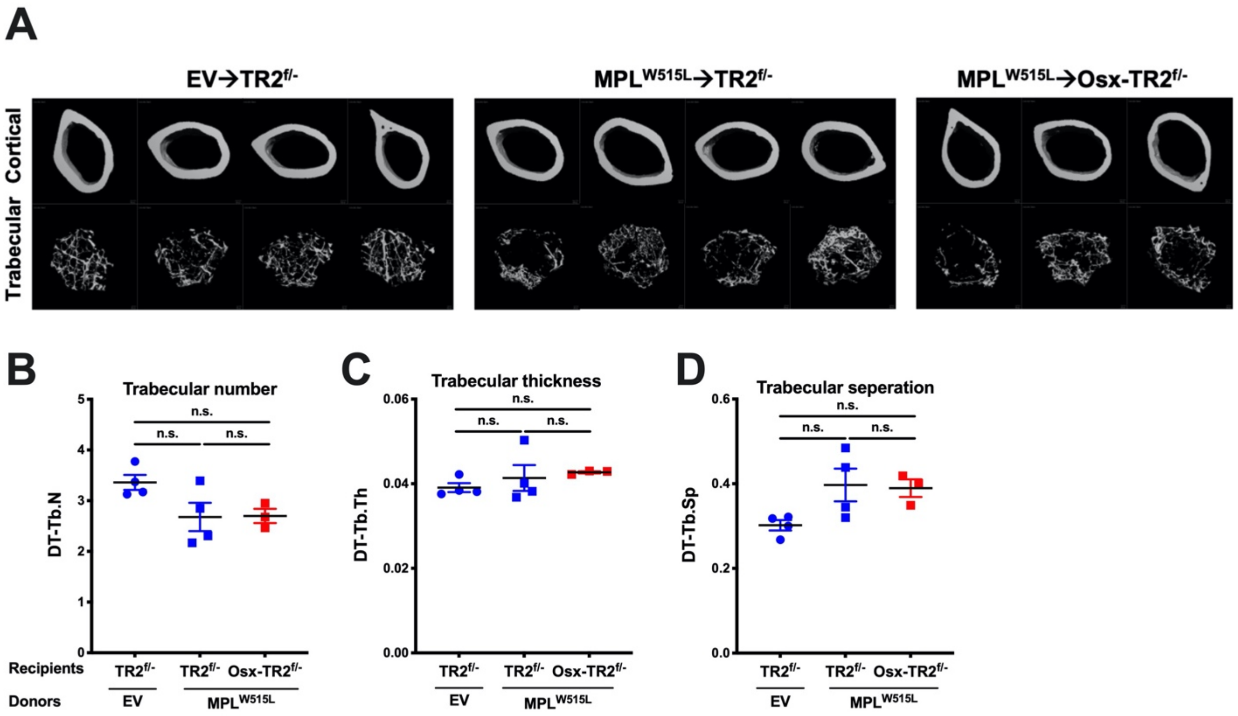

Supplementary Figure 4. Micro-CT analysis shows no significant changes in trabecularization in *MPL*^{W515L} induced MPN. (**A**) Radiographs of cortical and trabecular regions in femurs of *Tgfbr2*$^{f/-}$ (TR2$^{f/-}$) and *Osx-Cre; Tgfbr2*$^{f/-}$ (Osx-TR2$^{f/-}$) mice transplanted with *MPL*^{W515L}-transduced HSPCs. EV: empty vector. (**B**) Trabecular number, (**C**) trabecular thickness and (**D**) trabecular separation quantified from micro-CT radiographs. Data represent the mean ± SEM. Significance calculated by one-way ANOVA.

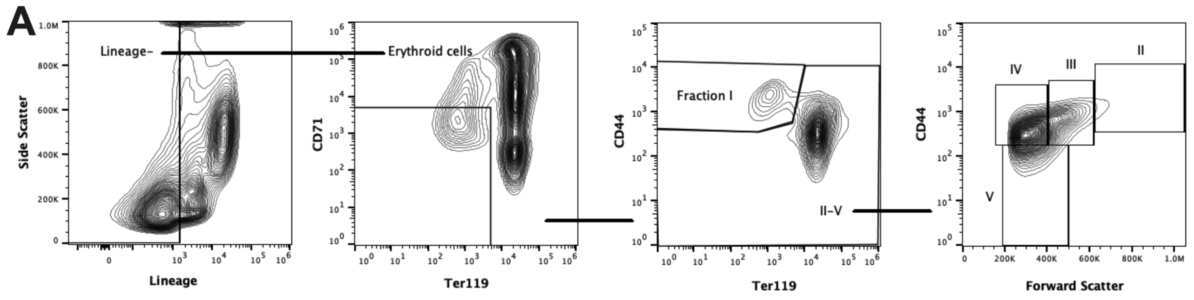

Supplementary Figure 5. Erythroid progenitor analysis. (**A**) Representative flow cytometry plots showing the gating strategy to identify the following erythroid fractions: I: proerythroblasts ($CD44^+$, $Ter119^{low}$ $lineage^-$ cells); II: basophilic erythroblasts ($CD44^+$ $Ter119^{high}$ FSC^{hi} $lineage^-$ cells); III: polychromatic erythroblasts ($CD44^+$ $Ter119^{high}$ FSC^{mid} $lineage^-$ cells), IV: orthochromatic erythroblasts ($CD44^+$ $Ter119^{high}$ FSC^{low} $lineage^-$ cells).

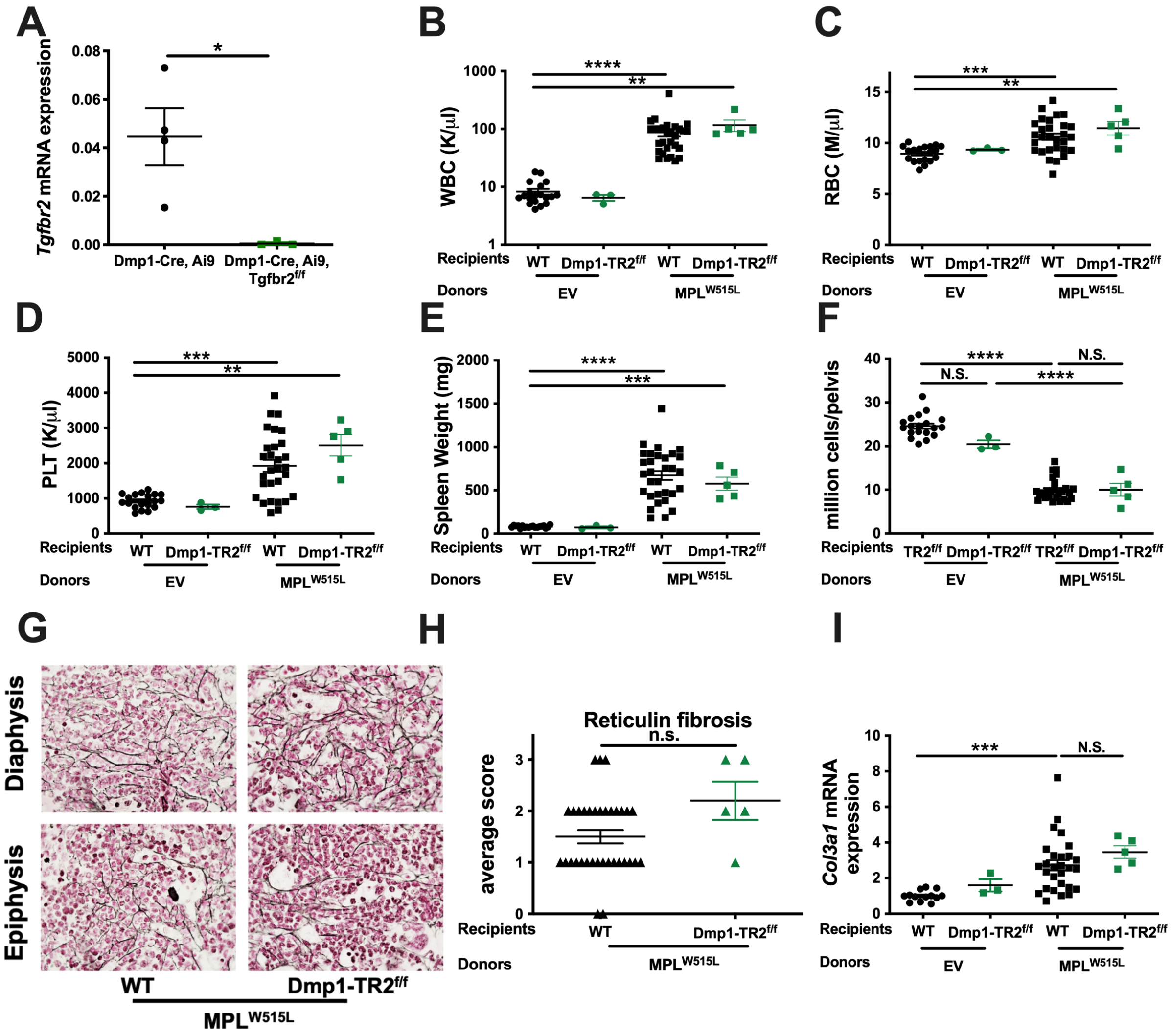

Supplementary Figure 6. TGF-β signaling in *Dmp1-Cre* targeted osteolineage cells is not required for the induction of myelofibrosis by *MPL*^{W515L}. (**A**) Relative *Tgfbr2* mRNA expression in lineage^{-} tdTomato^{+} bone marrow stromal cells sorted from *Dmp1-Cre; Tgfbr2*$^{f/f}$; *Ai9* mice. (**B-H**). *MPL*^{W515L}-transduced HSPCs were transplanted into *wildtype* (WT) or *Dmp1-Cre; Tgfbr2*$^{f/f}$ (Dmp1-TR2$^{f/f}$) mice. (**B**) White blood cell (WBC) count, (**C**) red blood cell (RBC) count, (**D**) platelet (PLT) count, (**E**) spleen weight and (**F**) bone marrow cellularity per pelvis 4 weeks after transplantation. (**G**) Representative photomicrographs of femur sections stained for reticulin. (**H**) Average score of fibrosis grading in the diaphysis and epiphysis. (**I**) mRNA expression levels of Collagen 3 (*Col3a1*) relative to β-actin mRNA in total bone marrow. Mice were analyzed 4 weeks post-transplantation. Data represent the mean ± SEM. *p<0.05, **p<0.01, ***p<0.001, ****p<0.0001 by Student's t-test (A), one-way ANOVA (B-F, I) or Kruskal-Wallis test (H).

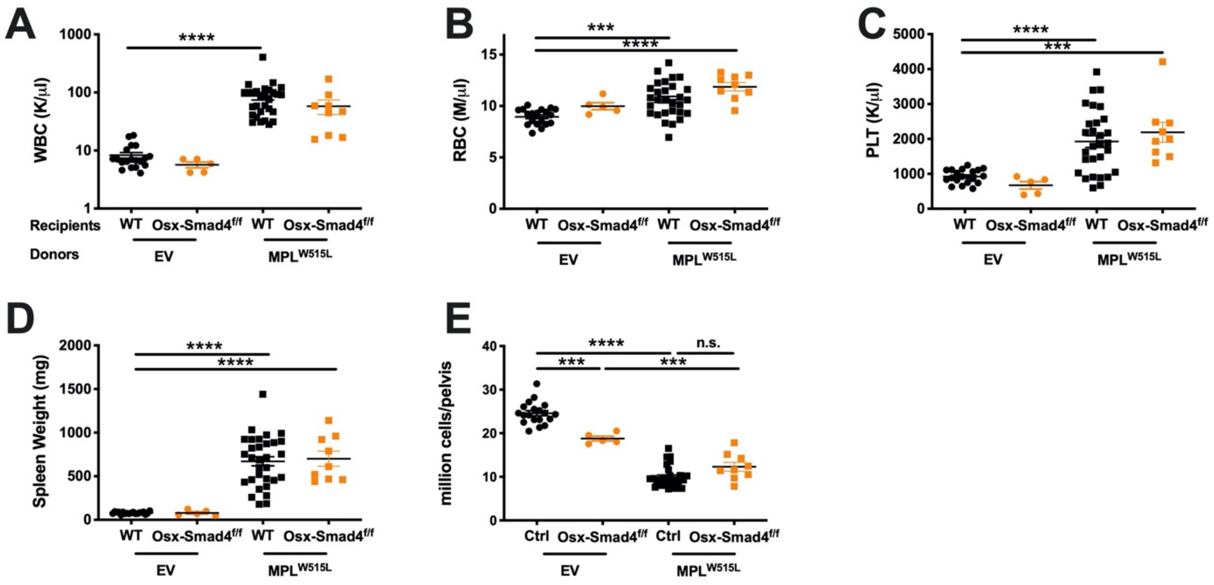

Supplementary Figure 7. Canonical (SMAD4-dependent) TGF-β signaling in *Osx-Cre* targeted mesenchymal stromal cells is not required for the development of the myeloproliferative phenotype by *MPL*^{W515L}. *MPL*^{W515L}-transduced HSPCs were transplanted into *wildtype* (WT) or *Osx-Cre; Smad4*$^{f/f}$ (Osx-Smad4$^{f/f}$) mice. Shown is the (**A**) White blood cell (WBC) count, (**B**) red blood cell (RBC) count, (**C**) platelet (PLT) count, (**D**) spleen weight and (**E**) bone marrow cellularity per pelvis 4 weeks after transplantation. Data represent the mean ± SEM. ***p<0.001, ****p<0.0001 by one-way ANOVA.

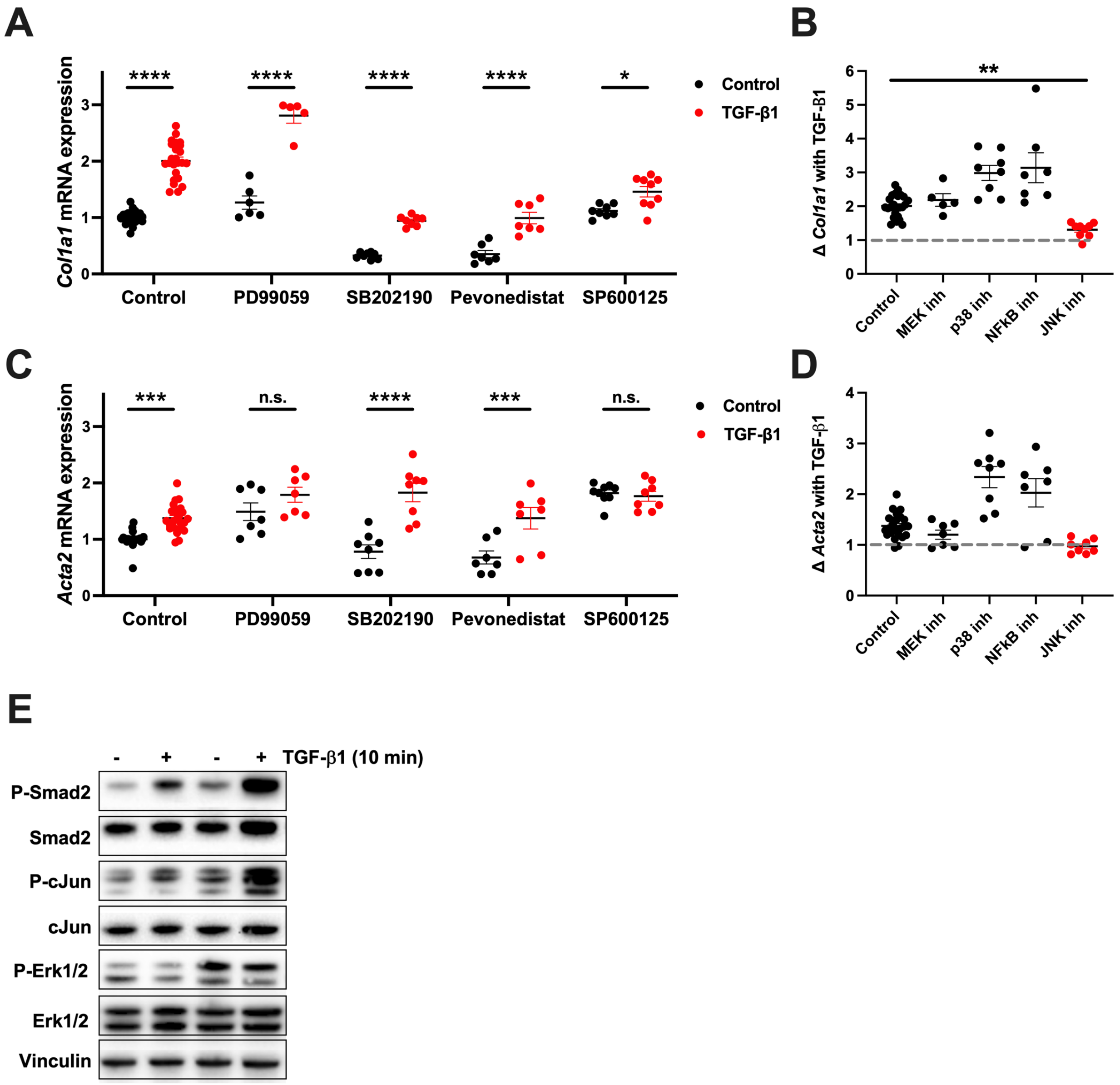

Supplementary Figure 8. *Col1a1* and *Acta2* mRNA expression in cultured MSCs treated with kinase inhibitors and TGF-β1. (**A, C**) Relative mRNA expression of *Col1a1* (A) and *Acta2* (C) in *wildtype* MSC cultures treated with TGF-β1 (10 ng/mL) and the MEK inhibitor PD99059 (20 μM), p38 inhibitor SB202190 (20 μM), and NFκB inhibitor pevonedistat (1 μM). (**B,D**) Fold change of *Col1a1* (B) and *Acta2* (D) expression after TGF-β treatment in each condition. (**E**) Immunoblot showing expression of the indicated protein (P-Smad2/P-cJun/P-Erk1/2: phosphorylated Smad2, cJun, or Erk1/2) in culture MSCs treated with vehicle alone or TGF-β1 (10 ng/mL) for 10 minutes. Data represents the mean ± SEM. *p<0.05, **p<0.01, ***p<0.001, ****p<0.0001 by two-way ANOVA (A, C) and one-way ANOVA (B).

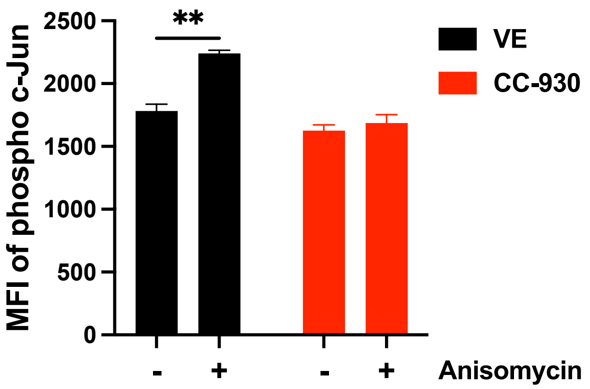

Supplementary Figure 9. CC-930 blocks JNK signaling activation *in vivo*. Mice were treated with 2 doses of CC-930 and c-Jun phosphorylation measured by flow cytometry in lineage negative (CD45, CD11b, Gr-1, CD3, B220, Ter119) bone marrow cells. Anisomycin (25 µg/mL, 15 mins) was used to induce JNK activation. VE: vehicle control. Data represents the mean ± SEM. **p<0.01 by two-way ANOVA.

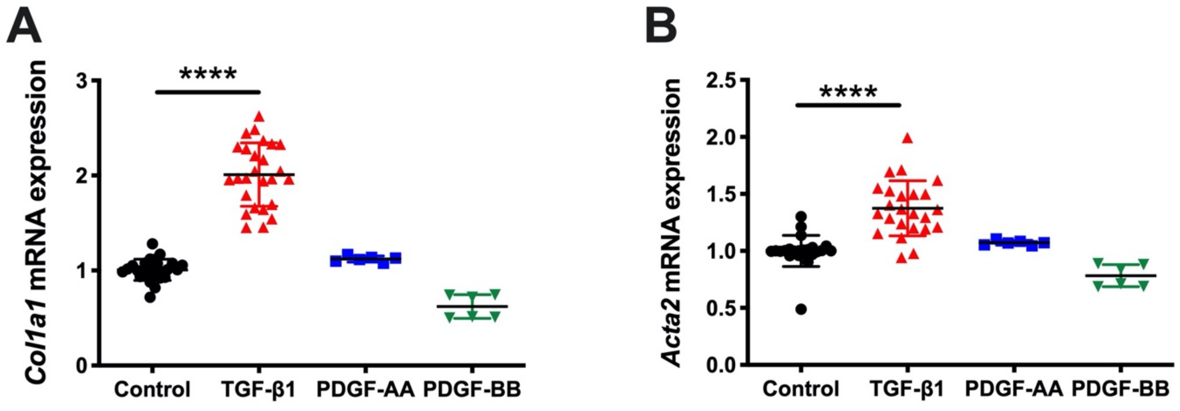

Supplementary Figure 10. *Col1a1* and *Acta2* mRNA expression in cultured MSC treated with TGF-β1 or PDGFs. (**A-B**) Relative mRNA expression of *Col1a1* (A) and *Acta2* (B) in *wildtype* MSC cultures treated with 10 ng/mL of TGF-β1, PDGF-AA or PDGF-BB for 72 hours. Data represent the mean ± SEM. ****p<0.0001 by one-way ANOVA.
